# Applying different emotion regulation strategies while providing negative feedback: Introduction of a new laboratory protocol

**DOI:** 10.1016/j.mex.2020.101162

**Published:** 2020-11-26

**Authors:** Christian L. Burk, Bettina S. Wiese

**Affiliations:** RWTH Aachen University, Jaegerstrasse 17-19, Aachen D-52056, Germany

**Keywords:** Method article, Laboratory protocol, Instructions, Negative feedback, Emotion regulation

## Abstract

One aim of creating a new laboratory protocol was to investigate stress responses while being confronted with a work-related task, that is, having to provide negative feedback. It was central to the development of the scenario to make potential testosterone and cortisol responses measurable. The first part of the protocol comprises the introduction to the cover story, that is, being a member of the assessors’ team as part of a larger assessment center program aiming to estimate the proficiency of students prior to their entry into professional life. Watching a video of one of the assessment center's candidates and having to assess his performance in a self-presentation task was introduced to personally involve participants in the feedback conversation they had to conduct with the same candidate later on.

A second aim was to introduce an experimental manipulation in the form of instructions and brief tutorials regarding different emotion regulation strategies to apply. Participants were randomly assigned to one out of four conditions: expressive suppression (keeping a neutral expression); cognitive reappraisal (staying task-oriented and emotionally distanced); affect utilization (moving towards and using emotions); or control condition. Distinguishing these ways to regulate one's emotions enabled us to reveal differential hormonal stress responses: Applying either cognitive reappraisal or affect utilization strategies alleviated temporary testosterone declines compared with the other two conditions.

This method article contains details regarding the procedure as well as the following documents in their original wording:

• Introduction to the cover story (being a member of the assessors’ team, observation of the attendant’s self-presentation, assessment dimensions)

• Slides and audio instructions regarding experimental conditions (how to regulate emotions)

• Documents handed out to assist participants in conducting the feedback conversation

## Graphical abstract

**Figure:**
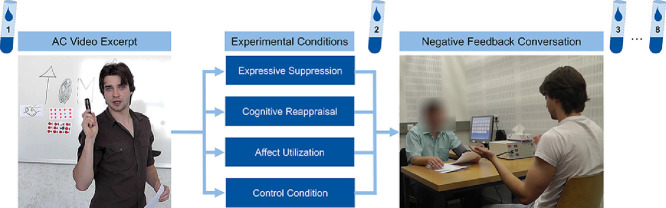

Specifications table

**Table:** 

Subject Area:	Psychology
More specific subject area:	*Emotion; I&O Psychology*
Method name:	*Laboratory Protocol: Emotion Regulation while Providing Negative Feedback*
Name and reference of original method:	Burk, C. L., & Wiese, B. S. (2021). How to alleviate the agony of providing negative feedback: Emotion regulation strategies affect hormonal stress responses to a managerial task. Hormones and Behavior, 127, 104868. https://doi.org/10.1016/j.yhbeh.2020.104868
Resource availability:	n/a

*Method details

## Overview: experimental procedure

**Fig. 1:**
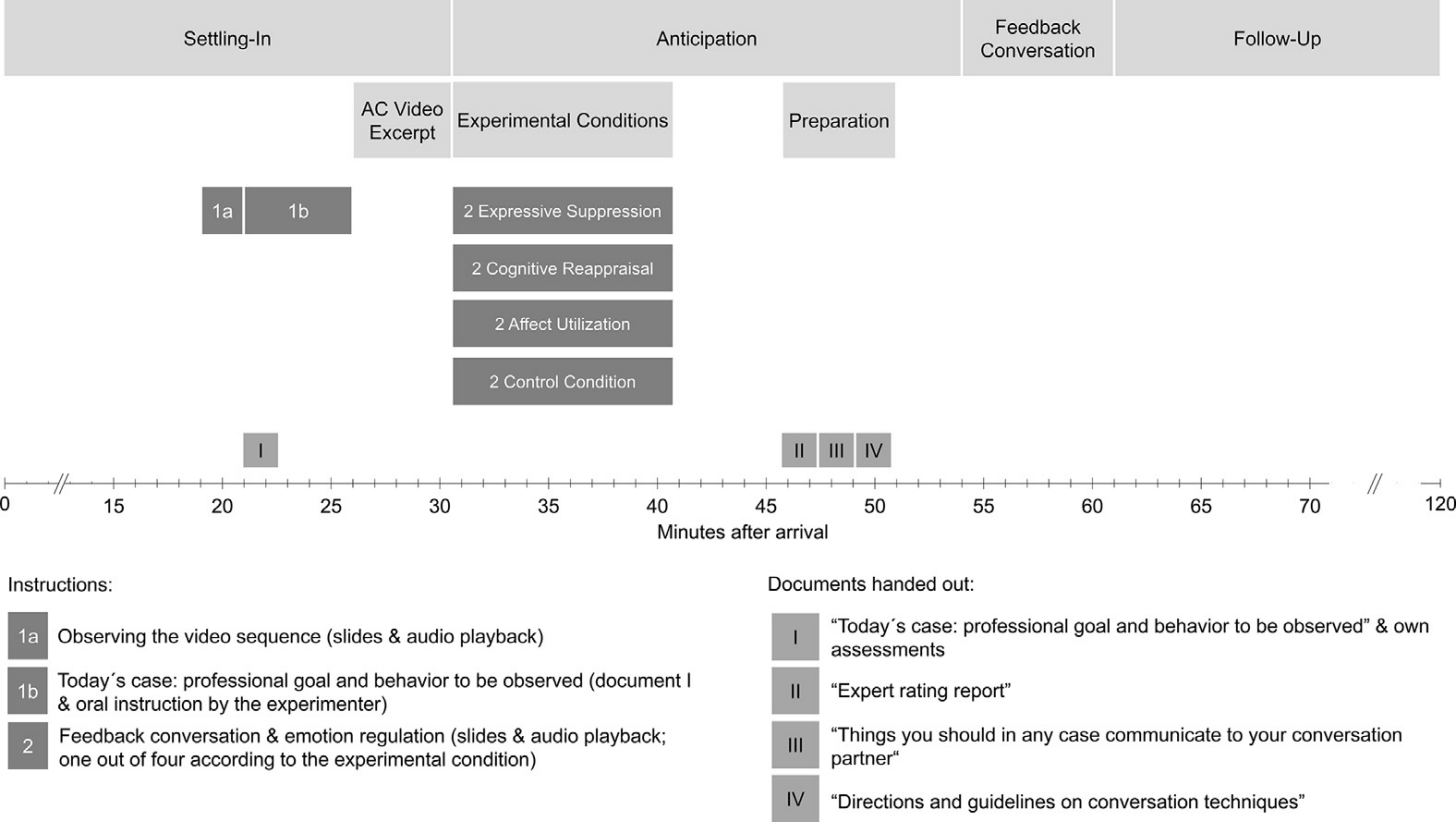
Simplified procedure of the laboratory session.

Fig. 1 provides an overview of the central part of the sessions’ procedure. For a complete description of the procedure, please refer to Burk & Wiese (2021). During a settling-in period, participants received information about the procedure, filled in questionnaires, and read magazines.

Beginning with minute 19, participants were introduced to the cover story and were instructed to watch a video excerpt from the assessment center showing the candidate they were going to conduct a feedback conversation with later on. This phase of the procedure is described in Section 2.

Beginning with minute 30.5, participants were instructed how to conduct the feedback conversation. Depending on the experimental condition the respective participant was assigned to, one out of four versions of instruction 2 was presented. These instructions addressed directions on how to regulate emotions during the conversation. These instructions are given in Section 3.

Four documents were handed out to assist participants in conducting the feedback conversation. The first of them (document I) was a written version of instruction 1b (handed out at minute 21) that introduced the candidate and his professional goal to the participant. In addition, document I contained information about the self-presentation task in question, directions on how to rate the candidate's behavior, and a corresponding rating sheet. These assessments were thought to serve as one source of the feedback to be provided afterwards. The content of document I is given in Section 3.2.

The ratings made by the participants were supplemented by document II, an expert rating report. Document III comprised a summary of the things the participant should in any case communicate to the conversation partner. This summary refers to the instructions all participants uniformly received during the opening and final parts of instruction 2. Finally, document IV summarized directions and guidelines on conversation techniques. As these directions referred to specific emotion regulation strategies, one out of four versions of document IV was handed out depending on the actual experimental condition. Documents II, III, and IV are displayed in Section 4.

After the anticipation period, including six minutes of preparation, the actual feedback conversation took place, which lasted seven minutes. During the conversation, the participants’ primary task was to explain the results of the self-presentation task with reference to their own assessments (document I), and the expert rating report (document II). Following the guidelines in document III, participants had to communicate scores on six dimensions, to recite experts’ comments, and to ask questions regarding the candidate's self-perception, reflection of the results, and conclusions concerning the candidate's professional future. Participants’ secondary task was to deal with a series of unpleasant emotions the professional actor exhibited in his role as the candidate.

Subsequently, a follow-up phase of approximately 60 min was established to depict the hormonal recovery.

For a complete description of the saliva sampling procedure, please refer to Burk & Wiese (2021). Table 1 provides details regarding the timing of the saliva samples’ collection with reference to the crucial phases of the laboratory protocol.

**Table 1:** Timing of eight saliva samples with reference to the phases of the laboratory protocol.

Saliva sample no.	1	2	3	4	5	6	7	8
Minutes from arrival	12.25	40.75	61	71	81	91	101	111
Minutes from starting to watch the AC video excerpt		14.75	35	45	55	65	75	85
Minutes from instruction 2 (feedback conversation and emotion regulation)		10.25	30.5	40.5	50.5	60.5	70.5	80.5
Minutes from starting the preparation period			15.25	25.25	35.25	45.25	55.25	65.25
Minutes from starting the feedback conversation			7	17	27	37	47	57
Minutes from end of the feedback conversation			0	10	20	30	40	50

Please note that all slides and documents provided in this method article comprise translations of the German original materials used by Burk & Wiese (2021).

## Cover story: introduction to the conversation partner and observation of the video sequence

After settling in, participants were introduced to the cover story, that is, they were member of the assessors’ team within a development program for young graduates.

### Instruction 1a: “Observing the video sequence”

In the first part of instruction 1 (instruction 1a; see Table 2), participants were introduced to the assessment center. They were informed that today's session was part of a larger development program aiming to assess the proficiency of students prior to their entry into professional life. They were told that subsequently they were going to watch a video recording of one participant in the program while he was performing in a self-presentation exercise as part of a longer AC. Instruction 1a was presented through an on-screen presentation including an audio playback.

**Table 2:** Instruction 1a: “Observing the video sequence” (slides & audio playback).

Slide No.	Text Displayed on Slide	Audio Playback During Presentation
1	**Observing the Video Sequence** **Your upcoming task:** Watching an excerpt of an assessment center (self-presentation task)	In a few moments, it will be your task to watch an excerpt of an assessment center via video. This recording was produced in one of the recent weeks. The video shows a person while performing a self-presentation task. Later during today's experiment, you will conduct a short conversation with just that person. For every candidate, the tasks of the assessment center were custom-tailored depending on the desired professional goal in question.
2	**Aim of the assessment center:** To determine the fit between individual strengths and the requirements of the respective professional goal	The aim of the individual assessment center was to provide participants with selective information regarding the fit between their strengths and the requirements of their specific professional goal. This is meant to help students in their last academic year and graduates to find their way while making occupational choices. Getting on the right occupational track not only needs specialized knowledge of one's subject but also talent and personality that fit to a certain profession. This you can hardly learn at universities.
3	Requirement analysis with representatives of relevant target companies: **The two most crucial behavioral domains** for the target job were identifiedYou will receive detailed information (target job, behavioral domains in question) about the present case from the experimenter	With the help of representatives of relevant target companies, we developed a requirement analysis for every desired occupation the candidates named. For every target job, we identified two behavioral domains that proved to be the most crucial for a successful career entry. Now, the experimenter will hand out information to you concerning which target job will be addressed in the present case. In addition, you will learn more about the two behavioral domains that resulted from the specific requirement analysis. Beyond that, you will get further information about the candidate's instructions regarding the task that you will deal with today. Please remove the headphones for a while now and listen to the experimenter.

### Instruction 1b: “Today's case: professional goal and behavior to be observed”

**Fig. 2:**
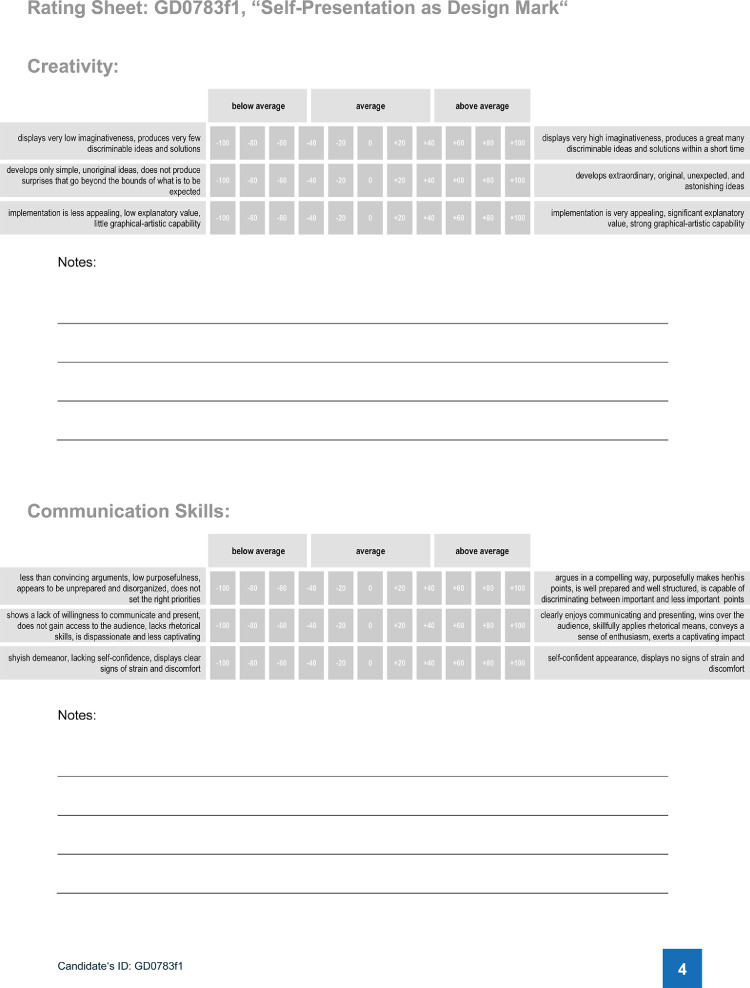
Document I, page 4 (instruction 1b).

Instruction 1b explicitly addressed the candidate that the participant was about to meet that day. This instruction was delivered in the form of document I, which was read aloud by the experimenter. Document I contained four parts: On the first page, participants were introduced to today's candidate, his subject, his professional goal, and the two demands to deal with (see Table 3, page 1). The second page displayed the instructions the candidate received for performing his self-presentation task (see Table 3, page 2). On page 3, participants were instructed which behavioral dimensions they should rate while watching the video (see Table 3, page 3). Page 4 comprised the rating sheet to be completed by the participant during the observation (Fig. 2).

**Table 3:** Document I, pages 1 to 3 (instruction 1b).

Page	Text
1	Candidate's ID: GD0783f1 Subject area: graphic design Professional goal: graphic designer in a marketing agency Crucial demands: creativity, communication skills **I. Today's Case: Professional Goal and Behavior to be Observed** Today's case will deal with **a male prospective graduate in graphic design** who called becoming **an art director in a marketing agency** his career goal. Two core demands have been named to successfully enter this desired position: **creativity** **communication skills.**
2	**Candidate's Instructions Regarding the Task “Self-presentation as Design Mark”** The excerpt from the assessment center that you will observe in a moment is about the following task: ***Self-presentation as Design Mark*** *The marketing agency you apply to is a strategic business consultancy that makes its customers develop an image and supports them regarding their brand management.* *Now it is about your personal trademark motto that summarizes you as a person as well as the characteristics of your personality. Imagine yourself as being a trademark. What does it stand for?* *Please produce a collage that illustrates the world of your trademark – what you stand for, what you represent, what you are good at. You may want to use some decent metaphors to get to the heart of your trademark. In addition, please develop a logo representing your trademark that illustrates your strengths and the atmosphere in your trademark's world.* *Provide proof of your first-class creativity and graphical-artistic capability. Convince your audience with passion and communication skills – just as you would do when presenting a trademark design to one of the agency's customers.* *Now, you have 15 min to prepare for the presentation. You may use all the tools from the presentation case as well as the whiteboard or flip chart.* *Within exactly four minutes, please present your ideas to us and describe how you developed your logo and what it is trying to say.*
3	**Your Instructions Regarding the Observation of the Task “Self-presentation as Design Mark”** Please observe the self-presentation of the candidate very carefully. Imagine you are a member of the consultancy that is responsible for analyzing the performance of the candidate. Together with other experts that have already observed the candidate's behavior before, it is your task to evaluate whether the applicant fully meets the most demanding requirements of **remarkable creative potential and originality as well as** **above-average communication skills, persuasiveness, and presentation skills.** Where applicable, make a note of distinctive features of the talk: for instance, significant attainments or mistakes/deficiencies of the candidate. Please gear your judgements towards what you would expect from an average graduate of the same subject. If the candidate meets your expectations, no more and no less, place your rating in the middle range of the scale. The better the candidate performs, the more you should adjust your rating towards the right end of the scale (up to a maximum of +100). If you have the impression of a below-average performance, adjust your rating towards the left (minimum: −100).

## Experimental condition: instructions for conducting the feedback conversation and for regulating emotions

Beginning with minute 30.5, participants were instructed how to conduct the feedback conversation. The opening and final parts of these instructions were uniformly presented to every participant (see Section 3.1). In between, a section was inserted depending on the experimental condition the respective participant was assigned to (Sections 3.2 to 3.5).

### Instructions uniformly presented in all conditions

The opening (see Table 4) and final (Table 5) parts of instruction 2 were identically presented to all participants. These parts contained the announcement of the upcoming feedback conversation, listed the documents that were available to assist the participant while providing feedback, and provided general information about the emotional content of such a conversation and directions on how to behave.

**Table 4:** Opening part of instruction 2 (uniformly presented in all conditions).

Slide No.	Text Displayed on Slide	Audio Playback During Presentation
1	Feedback Study Instruction: Feedback conversation	There now follow instructions for you concerning the feedback conversation.
2	In a few minutes, will ask the candidate to enter this room. You have just observed this candidate in the video recording and made judgements on his speech. Your task will be to provide performance feedback within a seven-minute period.	In a few minutes, we will ask the candidate to enter this room. You have just observed this candidate in the video recording and made judgements on his speech. Your task will be to provide performance feedback within a seven-minute period.
3	The following documents may assist you while providing feedback: I. Your own assessments from the document “Today's case: professional goal and behavior to be observed” II. Expert rating report III. “Things you should in any case communicate to your conversation partner” IV. “Directions and guidelines on conversation techniques”	The following documents may assist you while providing feedback: 1. Your own assessments you delivered while watching the video clip of the assessment center task. 2. A report with ratings concerning the assessment center sequence prepared by our experts. 3. A list with the things you should in any case communicate to your conversation partner. 4. A list comprising directions and guidelines on conversation techniques.
4	Please note: • You are a member of the assessment center team • Please do not mention being a voluntary study participant • Please use phrases like “we,” “our team,” and “our raters” • Please do not distance yourself from either the tasks or the results of the assessment center. Instead, please state that you completely support the procedure	Please note: Your conversation partner assumes that you are a member of the assessment center team. Hence, please do not mention being a voluntary study participant. Whenever talking about the assessment center's procedure or the judgements, please use phrases like “we,” “our team,” and “our raters.” These phrases should always include yourself as part of the team. Please do not distance yourself from either the tasks or the results of the assessment center. Instead, please state that you completely support the procedure.
5	Please keep in mind that it is important to your conversation partner to get precise information concerning his or her aptitude for his/her vocational ambitions. Hence, there is a lot at stake for your conversation partner.	Please keep in mind that it is important to your conversation partner to get precise information concerning his or her aptitude for his/her vocational ambitions. Hence, there is a lot at stake for your conversation partner.
6	Please be prepared for the fact that the conversation may take a quite emotional course. A consistently positive feedback may give great pleasure to your conversation partner. Please note that your conversation partner might experience disappointment if his or her results are not uniformly positive.	Please be prepared for the fact that the conversation may take a quite emotional course. A consistently positive feedback may give great pleasure to your conversation partner. Please note that, as may also be the case, your conversation partner might experience disappointment if his or her results are not uniformly positive.
7	In this type of conversation, it is not unusual that pleasant or unpleasant emotions occur – either on the side of your conversation partner or, in response, on your side.	In this type of conversation, it is not unusual that pleasant or unpleasant emotions occur – either on the side of your conversation partner or, in response, on your side. People that regularly conduct such conversations for professional reasons often receive detailed directions from their employer on how to behave if things become emotional. We are also going to supply you with directions and guidelines on how to conduct yourself ideally during the conversation.

**Table 5:** Final part of instruction 2 (uniformly presented in all conditions).

Slide No.	Text Displayed on Slide	Audio Playback During Presentation
22/ 25/ 20/ 17	It is very important that you follow our directions and tips concerning which conversation techniques to apply. It is equally important that you pass on to your counterpart the information that you will find in the list “Things you should in any case communicate to your conversation partner.”	It is very important that you follow our directions and tips concerning which conversation techniques to apply. It is equally important that you pass on to your counterpart the information that you will find in the list “Things you should in any case communicate to your conversation partner.”
23/ 26/ 21/ 18	The experimenter will observe your behavior during the conversation and will evaluate how accurately you follow our directions. In addition, experts on conversation techniques will judge the video recording of the conversation later on. Please keep in mind: The more accurately you follow the directions, the greater the chance of being among the 10% best participants, which earns a bonus afterwards.	The experimenter will observe your behavior during the conversation and will evaluate how accurately you follow our directions. In addition, experts on conversation techniques will judge the video recording of the conversation later on. Please keep in mind: The more accurately you follow the directions, the greater the chance of being among the 10% best participants, which earns a bonus afterwards.
24/ 26/ 21/ 18	The experimenter will now hand over the documents that may assist you while providing feedback. After that, we will conduct the next mood and blood pressure measurements, and will present the next picture series. Following this, you will have the opportunity to become familiar with the documents and to prepare for the conversation.	The experimenter will now hand over the documents that may assist you while providing feedback. After that, we will conduct the next mood and blood pressure measurements, and will present the next picture series. Following this, you will have the opportunity to become familiar with the documents and to prepare for the conversation.

### Expressive suppression condition

If assigned to the expressive suppression condition, instructions to keep a neutral facial expression and a tutorial about mimic movements were inserted following the opening part of instruction 2. Instructions to suppress emotional expressions were partly adapted from Gross (1998). Table 6 displays the presentation slides and the wording of the audio track.

**Table 6:** Instruction 2: Insertion for the expressive suppression condition.

Slide No.	Text Displayed on Slide	Audio Playback During Presentation
8	**Hints and Tips Concerning the Conversation** Retain a neutral facial expression, so that nobody can observe any emotion.	Helpful hints and tips for you on how to approach the conversation Retain a neutral facial expression, so that nobody can observe any emotion.
9	• In the event that you notice any emotions arising inside yourself during the feedback conversation, please take care not to reveal, but rather hide these. • Dissemble any feelings you might have. • Please behave in a way that means no one watching from the sidelines can notice you feeling anything at all.	In the event that you notice any emotions arising inside yourself during the feedback conversation, please take care not to reveal, but rather hide these. Dissemble any feelings you might have. Please behave in a way that means no one watching from the sidelines can notice that you are feeling anything at all.
10	• Do not respond to possible emotional expressions on the part of your conversation partner. • Do not express any emotions on your part. • Keep your facial muscles under control and do not move them.	Please do not respond to possible emotional expressions on the part of your conversation partner, and do not express any emotions on your part. An important component of this is keeping your facial muscles under control. Please do not move your facial muscles as they can reveal what is going on inside you emotionally. In order to assist you in implementing this, the following provides an introduction to three common emotions and how they disclose themselves on your face.
11	Example No. 1: Anger [Video: Anger Expression]	Here, you can see a person getting angry. Please take special note of how he is knitting his eyebrows and how he presses his lips together.
12	Anger • do not knit your eyebrows • do not open your eyes widely • do not tighten or press your lips together • do not clench your teeth • keep the muscles of the jaw and neck relaxed	Please take care not to frown or knit your eyebrows so that deep wrinkles appear between them. Please do not raise your upper eyelids; widely opening your eyes and staring will make anger recognizable. Please take care not to tighten or press your lips together; it would be undesirable for one to observe that the redness of your upper lip had faded or your lips were pressed together. Please do not clench your teeth. Please keep the muscles of the jaw and neck relaxed. Muscular tension will reveal anger.
13	Anger Please bear your voice in mind: • even, natural pitch in your voice • do not increase amplitude • do not increase the pitch • do not increase the tempo	Moreover, please remember to retain an even, natural pitch in your voice. Increasing the amplitude, pitch, or tempo of your voice could be interpreted as a sign of anger.
14	Example No. 2: Joy [Video: Expression of Joy]	Here, a person displays joy. In particular, note the raising of the cheeks, the crow's feet wrinkles, and the lip corners pulled back.
15	Joy • Mouth: do not pull up your lip corners • do not let a furrow appear between your upper lip corners and your nasal wings • do not raise your cheeks, avoid crow's feet wrinkles	Please remember not to pull up your lip corners. Please do not let a furrow appear between your upper lip corners and your nasal wings. Take care not to raise your cheeks. In particular, avoid displaying crow's feet wrinkles at the outer eye corners or squinting.
16	Joy Please try not to show any other signs of joyful arousal: • fidgeting on your chair • leaning forward • increasing the amplitude or pitch of your voice	Moreover, please try not to show any other signs of joyful arousal: for instance, fidgeting on your chair, leaning forward towards your conversation partner, speaking with a loader voice or increasing pitch.
17	Example No. 3: Sadness/Disappointment/Pity [Video: Expression of Sadness]	Here, a person is sad, disappointed, or takes pity on someone. Pay attention to horizontal wrinkles on the forehead and depression of the lip corners.
18	Sadness/Disappointment/Pity • Mouth: do not pull down your lip corners • Eyes/forehead: avoid horizontal wrinkles on the forehead	Please take care not to pull down your lip corners. Even the slightest horizontal wrinkles on the forehead, which are produced by pulling the inner portion of the eyebrows upwards, are detected as a sign of sadness.
19	Sadness/Disappointment/Pity • do not let your shoulders droop • do not lower your eyes • do not allow your lips or chin to tremble	In addition, please take care not to display any other signs of sadness or pity: for instance, letting your shoulders droop, lowering your eyes, or allowing your lips or chin to tremble.
20	Please keep your facial muscles motionless to retain a neutral expression!	Please keep your facial muscles motionless to retain a neutral expression!
21	Please try to behave in accordance with our directions, instead of behaving in a way you would usually consider comfortable or fitting to your personal style.	Please try to behave in accordance with our directions, instead of behaving in a way you would usually consider comfortable or fitting to your personal style.

### Cognitive reappraisal condition

In the cognitive appraisal condition, participants were instructed to stay task-oriented and emotionally distanced. Participants were instructed to behave like neutral assessment professionals, always following the notion that detailed negative feedback helps the feedback receiver to obtain information and to improve their performance in the future. These instructions were partly adapted from Gross (1998). A tutorial addressed how to extract and communicate facts from the experts’ report sheet (see Table 7).

**Table 7:** Instruction 2: Insertion for the cognitive reappraisal condition.

Slide No.	Text Displayed on Slide	Audio Playback During Presentation
8	Hints and Tips Concerning the Conversation Adopt the neutral and distanced attitude of a professional assessor and provide as much factual information as possible. Try to think about the conversation in such a way that no emotions build up.	Helpful hints and tips for you on how to approach the conversation Adopt the neutral and distanced attitude of a professional assessor and provide as much factual information as possible. Try to think about the conversation in such a way that no emotions build up.
9	• do not regard the feedback situation as being a personal matter • role of a professional, psychological assessor • perceive the situation with a detached interest • retain an objective perspective, avoid committing yourself to possible emotions	Please do not regard the feedback situation as being a personal matter. Fulfill a role of a professional, psychological assessor and perceive the situation with a detached interest. Try, as much as possible, to retain an objective perspective and to avoid committing yourself to possible emotions on the side of your conversation partner.
10	• think objectively and analytically • do not consider it as personally or emotionally relevant • not involved emotionally, do not feel anything at all	Think about the conversation objectively and analytically rather than considering it personally or emotionally relevant. Try to think about the conversation in such a way that you are not involved emotionally and you do not feel anything at all.
11	Guidelines on Conversation TechniquesYou might tell yourself: • I simply provide feedback and have not been involved in the production of these results. • It does not have any further impact on me personally, whether the candidate is delighted or disappointed.	For example, you might tell yourself that you simply provide feedback concerning the results and have not been involved in the production of these results. Remember that you are not responsible personally for the compilation of the tasks or for the results. It does not have any further impact on you personally, whether the candidate is delighted or disappointed to hear about his or her results.
12	Guidelines on Conversation Techniques Note: • Your conversation partner has voluntarily attended the assessment center in order to learn more about his or her strengths and weaknesses. • Contribute to a professional vocational counseling by providing objective and detailed performance feedback. • the candidate will benefit from the feedback • Fostering self-awareness concerning possible weaknesses is a crucial step towards achieving an adequate professional orientation. „The most important thing about career choices is to know as much as possible about oneself – with a focus on both positive and negative aspects.”	Please note that your conversation partner has voluntarily attended the assessment center in order to learn more about his or her strengths and weaknesses as well as to promote his or her professional orientation. Professional vocational counseling should comprise objective and detailed performance feedback. In any case, the candidate will benefit from the feedback, even though it may not be exclusively positive. Fostering self-awareness concerning possible weaknesses is a crucial step towards achieving an adequate professional orientation. Adopt the neutral perspective: “The most important thing about career choices is to know as much as possible about oneself – with a focus on both positive and negative aspects.”
13	Guidelines on Conversation TechniquesAlways focus on the facts! • pay attention to the details of the expert rating sheet • express as many details as possible • try to memorize as much of the report's information as possible Tell yourself: “The figures speak for themselves!“	Always focus on the facts. Please pay attention to the details of the expert rating sheet and express as many details as possible. Try to memorize as much of the report's information as possible, so as to give account when prompted for it. Tell yourself – and maybe use these words during the conversation: “The figures speak for themselves!” In order to ensure that you will do well in regard to focusing on the facts, there now follows a short explanation of the expert rating report, which you will base the conversation on.
14	Information that may be extracted from the expert rating report	Each of the six dimensions displayed in the report contains the following information: At the top, one of the two main target variables, which turned out to be the most important as part of the requirement analysis, is given.
15	Information that may be extracted from the expert rating report	Below that, you will find the title of the assessment center task this report refers to.
16	Information that may be extracted from the expert rating report	On the right and left ends of the diagram, examples of typical behavior are given that persons with notably high (placed on the right) or low (placed on the left) values display in the respective dimension.
17	Information that may be extracted from the expert rating report	The bar represents the score of the actual candidate.
18	Information that may be extracted from the expert rating report	The middle of the scale depicts the average of all candidates. This point is labeled with “M” (for “mean”) and corresponds to a score of zero. Beginning with the middle of the scale, the candidate's bar stretches to the right (in the case of positive values) or left (in the case of negative values). Within the near range around zero, scores may be called “average.”
19	Information that may be extracted from the expert rating report	The further the bar reaches toward the right end, the more likely it is that you can apply the statement on the right to the candidate. These represent high scores in the respective dimension. As soon as the bar exceeds the second color grading – labeled “+1 SD” (for one standard deviation above the mean) – you can call the candidate's score “above average.” At the fourth grading, labeled “+2 SD,” you can call it “well above average.”
20	Information that may be extracted from the expert rating report	The further the bar reaches toward the left end, the more the statement on the left applies, representing a low score in the dimension. Exceeding “−1 SD” to the left, we begin by talking about “below average” scores; exceeding “−2 SD” we call them “well below average” scores.
21	Information that may be extracted from the expert rating report	Below the diagram, the results are summarized. First, each result is classified in reference to the whole group of candidates, as, for instance, average or below or above average.
22	Information that may be extracted from the expert rating report	Second, you will once more find the exact score averaged across all expert raters on a scale from −100 to +100.
23	Information that may be extracted from the expert rating report Please state such additional facts during the conversation in order to provide informative and objective feedback to your conversation partner!	Third, the percentile rank indicates the percentage of candidates that achieved a weaker score than, or the same score as, the actual candidate. For instance, a percentile rank of 50 would mean that just as many persons have been done better as those that have done worse than the actual candidate did. As regards the example displayed here on the slide, 94% of the candidates have performed weaker than, or equal to, the candidate in question; only 6% have performed even better. Please state such additional facts during the conversation in order to provide informative and objective feedback to your conversation partner!
24	Please try to behave in accordance with our directions, instead of behaving in a way you would usually consider comfortable or fitting to your personal style.	Please try to behave in accordance with our directions, instead of behaving in a way you would usually consider comfortable or fitting to your personal style.

### Affect utilization condition

In the affect utilization condition, participants were requested to take the conversation partner's perspective and to turn towards the emotions he displays. They received a tutorial about how to apply techniques in the sense of client-centered therapy (Rogers, 1951), that is, active listening, paraphrasing, and verbalization of emotions (see Table 8).

**Table 8:** Instruction 2: Insertion for the affect utilization condition.

Slide No.	Text Displayed on Slide	Audio Playback During Presentation
8	Hints and Tips Concerning the Conversation • Get an idea of the emotional content of the conversation. • Get involved with your own feelings as well as the feelings of your conversation partner. • Try to perceive all emotions.	Helpful hints and tips for you on how to approach the conversation Besides providing feedback regarding the results, please regard it as your duty to get an idea of the emotional content of the conversation. Please get involved with your own feelings as well as the feelings of your conversation partner. Try to perceive all emotions that potentially arise. Be mindful of the facial expression and the body language of the candidate so as to recognize her or his emotions. Also, pay attention to possible signs of arousal (for instance, trembling, fidgeting, blushing, or changes in pitch of the voice) as indicators of joy, anger, or sadness/disappointment.
9	Hints and Tips Concerning the Conversation • Put yourself in your conversation partner's position. • Actively turn towards the emotions your conversation partner displays by addressing them. • Express active interest.	Please try as best as you can to put yourself in your conversation partner's position. Imagine what you would feel in her or his place. Where appropriate, express what you would feel if you were her or him. You may also tell her or him about your own experiences with receiving positive or negative feedback. Please actively turn towards the emotions your conversation partner displays by addressing them. Express active interest.
10	Hints and Tips Concerning the Conversation • Someone watching you should be able to recognize that you have noticed any emotional nuance. • Make yourself an “expert” in every mood and emotion that emerges during the conversation.	Please respond in such a way that someone watching you would recognize that you have noticed any emotional nuance the candidate has expressed. An outside observer should be able to learn everything about the candidate's emotions just based on your expressions and without the need to observe the candidate her- or himself. Please try to make yourself an “expert” in every mood and emotion that emerges during the conversation. Please try as best as you can to memorize all the different emotions that appear, so that you can recall them when asked later on.
11	Please lend a sympathetic ear! • Give your conversation partner the feeling of being understood. • Do not judge her/him personally. • Listen acceptingly. • Show honest interest in her/his thoughts and feelings. • Signal your readiness to address your conversation partner's concerns by ○ nodding your approval ○ keeping eye contact ○ making affirmative sounds like “mhm, yes”	Please lend a sympathetic ear! Give your conversation partner the secure feeling of being understood. This can best be done by not judging her/him personally but listening acceptingly and showing honest interest in her/his thoughts and feelings. Signal your readiness to address your conversation partner's concerns by nodding your approval, keeping eye contact, and making affirmative sounds like “mhm, yes.”
12	Restate your conversation partner's remarks in your own words! • Consider thoughts and feelings. • Give the gist of what the candidate has said. • Observe your counterpart's reaction.	Restate your conversation partner's remarks in your own words! In particular, consider the thoughts and feelings of your counterpart. Right after you have given the gist of what the candidate has said, please observe your counterpart's reaction and make sure that she or he agrees with your summary.
13	Restate your conversation partner's remarks in your own words!The candidate says: “The assessment center task was an entirely new situation for me. I was quite excited.” You may reply: “I think I understand you. If one does not have extensive experience with tasks like that, it is sometimes not so easy to keep cool, right?” • you avoid misunderstandings • you signal appreciation and establish trust • you provide the opportunity to perceive and express feelings even clearer	Let us take an example: The candidate says: “The assessment center task was an entirely new situation for me. I was quite excited.” You may reply: “I think I understand you. If one does not have extensive experience with tasks like that, it is sometimes not so easy to keep cool, right?” With this, you recheck whether you have properly comprehended everything and you help avoid misunderstandings. Moreover, you signal appreciation and establish trust by demonstrating that you seriously concern yourself with her/his issues. Finally, you provide the opportunity to perceive and express her or his feelings even more clearly.
14	Bring up feelings directly! • Take particular care to identify hidden feelings. • Put the underlying feeling into words. Communicate to your conversation partner what feeling or mood you think you have discerned.	Bring up feelings directly!Please take particular care to identify hidden feelings even if they are not mentioned explicitly.Whenever you discover such an implicit feeling, please try the following: • do not repeat the whole statement • but rather put the underlying feeling into words Hence, communicate to your conversation partner what feeling or mood you think you have discerned.
15	Bring up feelings directly! • Try to identify what emotion might have hidden behind a statement. • Tell your conversation partner what emotion you have discovered. • Express your interpretation in the form of a question.	First, try to identify what emotion might have hidden behind a statement: for example, joy, excitement, anxiety, anger, uncertainty, disappointment, surprise, or confidence. Tell your conversation partner what emotion you have discovered behind her or his statement. Express your interpretation in the form of a question or declare that you have tried your best to guess the emotion accurately, but you are in doubt about it. Instead of using an assertive tone, request a reconfirmation. For clarification, we will provide three examples.
16	Bring up feelings directly: Example 1 “This task was like you made it just for me. I had hoped that something like this would be part of the assessment center.” You may answer: “**Am I right in saying** that you were **delighted** with the task and you felt **relieved**?” With this, you proclaim that you inferred delight and relief from the candidate's statement. Using the interrogative form and the beginning of the sentence “Am I right in saying,” you imply that you are uncertain.	Your conversation partner could, for example, say: “This task was like you made it just for me. I had hoped that something like this would be part of the assessment center.” You may answer: “Am I right in saying that you were delighted with the task and you felt relieved?” With this, you proclaim that you inferred delight and relief from the candidate's statement. Using the interrogative form and the beginning of the sentence “Am I right in saying,” you imply that you are uncertain.
17	Bring up feelings directly: Example 2 “This actually is a rather weak result. I really would not have expected to score that badly at this task.” Your possible response: “**I do not know exactly, but it seems to me** that you **promised** yourself a better result and may be **disappointed** now. **Is that right or am I totally wrong?”** You express the feelings you have perceived, namely hope and disappointment. The initial and closing sections signal uncertainty.	Let us take a second example: “This actually is a rather weak result. I really would not have expected to score that badly at this task.” Your possible response: “I do not know exactly, but it seems to me that you promised yourself a better result and may be disappointed now. Is that right or am I totally wrong?” You express the feelings you have perceived, namely hope and disappointment. The initial and closing sections signal uncertainty.
18	Bring up feelings directly: Example 3 “I do not agree with that. I cannot imagine what a task like this might have to do with my future professional life.” Your possible response: “**It feels to me as if** you felt **treated unfairly** to some extent. Are you **annoyed** about this task?”	A third and final example: “I do not agree with that. I cannot imagine what a task like this might have to do with my future professional life.” Your possible response: “It feels to me as if you felt treated unfairly to some extent. Are you annoyed about this task?”
19	Please try to behave in accordance with our directions, instead of behaving in a way you would usually consider comfortable or fitting to your personal style.	Please try to behave in accordance with our directions, instead of behaving in a way you would usually consider comfortable or fitting to your personal style.

### Control condition

In the control condition, participants received an approximate explanation of what some people might do to regulate their emotions. In contrast to the other conditions, participants did not receive any tutorial regarding how to deal with emotions but were asked to behave in a way that is most comfortable for them personally (see Table 9).

**Table 9:** Instruction 2: Insertion for the control condition.

Slide No.	Text Displayed on Slide	Audio Playback During Presentation
8	Hints and Tips Concerning the Conversation Please provide, if possible, professional feedback regarding the results of the assessment center task. Behave in the way you think is most comfortable for you.	Helpful hints and tips for you on how to approach the conversation Please provide, if possible, professional feedback regarding the results of the assessment center task. Because we realize that one can approach such a conversation in a variety of ways, we ask you to behave in the way you think is most comfortable for you.
9	In the following, we will present three common strategies for providing feedback. From this, you may extract tips you consider helpful for conducting the conversation.	In the following, we will present three common strategies for providing feedback. From this, you may extract tips you consider helpful for conducting the conversation.
10	Strategy No. 1Some feedback providers profit from retaining a neutral facial expression, so that nobody can observe any emotion. • not responding to possible emotional expressions on the part of their conversation partners • not expressing emotions • keeping one's facial muscles under control, because these may reveal what is going on inside a person emotionally	Some feedback providers profit from retaining a neutral facial expression, so that nobody can observe any emotion. Persons that follow such a strategy try not to respond to possible emotional expressions on the part of their conversation partners. They do not express emotions and take care to keep their facial muscles under control, because these may reveal what is going on inside a person emotionally.
11	Strategy No. 2Some feedback providers profit from • adopting the neutral and distanced attitude of a professional assessor • providing as much factual information as possible • trying to think about the conversation in such a way that no emotions build up	Some feedback providers profit from adopting the neutral and distanced attitude of a professional assessor and providing as much factual information as possible. These people try to think about the conversation in such a way that no emotions build up.
12	Strategy No. 2 • not regarding the feedback situation as being a personal matter • perceiving the situation with a detached interest • retaining an objective perspective and not committing oneself to possible emotions • acting objectively and analytically • not considering the conversation personally or emotionally relevant	These people do not regard the feedback situation as being a personal matter and perceive the situation with a detached interest. They try to retain an objective perspective and to avoid committing themselves to possible emotions on the side of their conversation partner. They act objectively and analytically and do not consider the conversation personally or emotionally relevant.
13	Strategy No. 2 • focusing on the facts • paying attention to the details of the expert rating sheet • expressing as many details as possible	Some people say it may help to always focus on the facts, and to pay attention to the details of the expert rating sheet. Some people will express as many details as possible.
14	Strategy No. 3Some feedback providers profit from • paying attention to emotions • getting involved with emotions • trying to perceive all emotions • trying to get an idea of the emotional content of the conversation	Some feedback providers profit from paying attention to emotions and getting involved with them. These people try to perceive all emotions that potentially arise and try to get an idea of the emotional content of the conversation.
15	Strategy No. 3 • putting oneself in the conversation partner's position • imagining what one would feel in the counterpart's place • actively turning towards the emotions the conversation partner displays: addressing, repeating, interpreting emotions	Such a person will try as best she or he can to put her-/himself in her/his conversation partner's position. Such a person might imagine what she or he would feel in the counterpart's place. Some people actively turn towards the emotions their conversation partners display by addressing these, repeating emotional content, and drawing emotional conclusions.
16	Let us summarize the strategies we have just presented: 1. keeping a neutral expression, 2. adopting the distanced attitude of a professional assessor, and 3. making oneself an “expert” in emotions Please note: No strategy is better or worse than any other. Just follow any strategy that works best for you personally.	Let us summarize the strategies we have just presented: 1. keeping a neutral expression, 2. adopting the distanced attitude of a professional assessor, and 3. making oneself an “expert” in emotions Please note: No strategy is better or worse than any other. Just follow any strategy that works best for you personally.

## Documents handed out to assist participants in conducting the feedback conversation

The first of the four documents handed out was a written version of instruction 1b and the participants’ own assessments regarding the video sequence (see Section 3.2).

Documents II (expert rating report), III (things to communicate), and IV (directions and guidelines on conversation techniques) are described in the following sections.

### Document II: “Expert rating report”

**Fig. 3:**
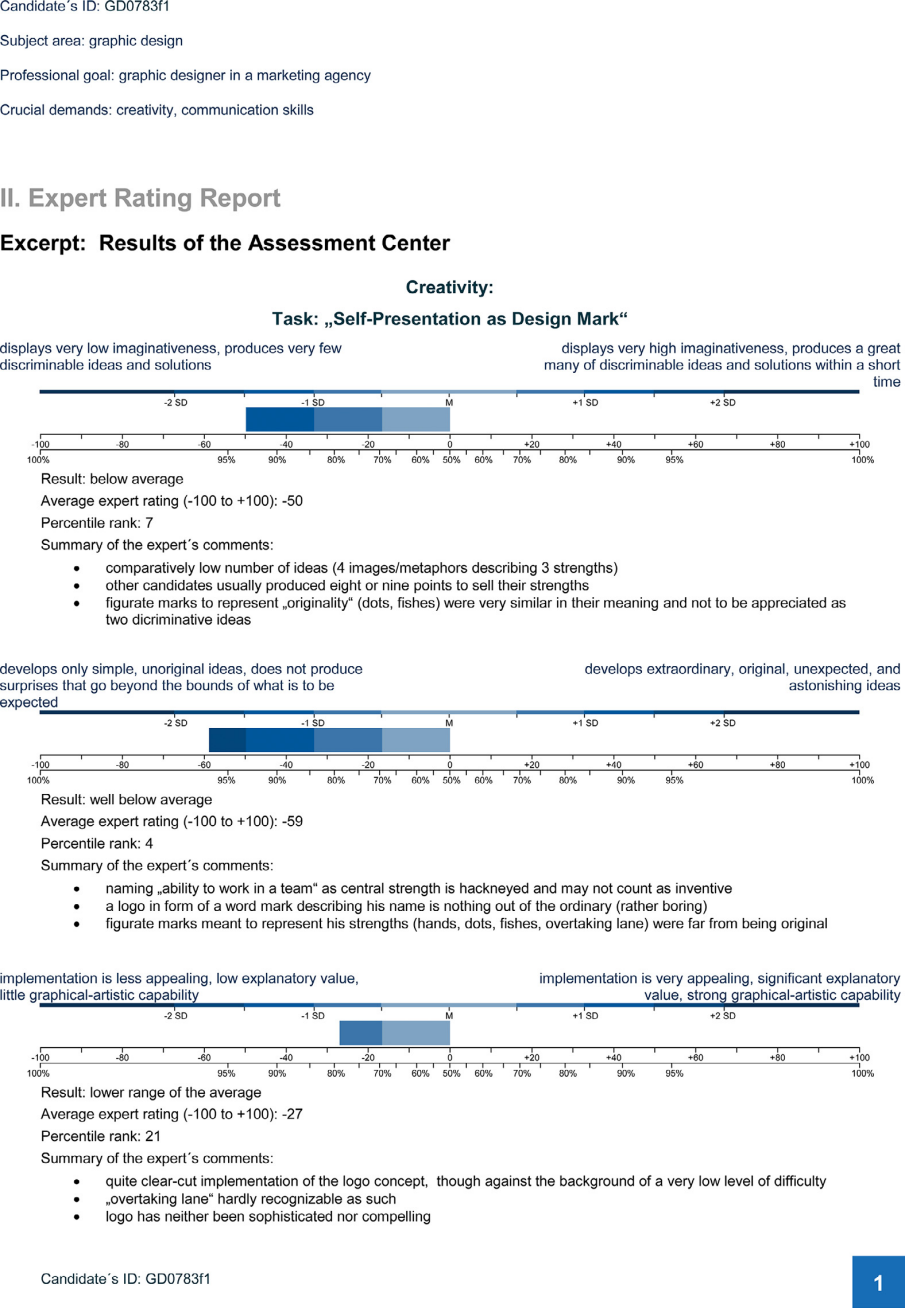
Document II: Expert rating report, page 1.

**Fig. 4:**
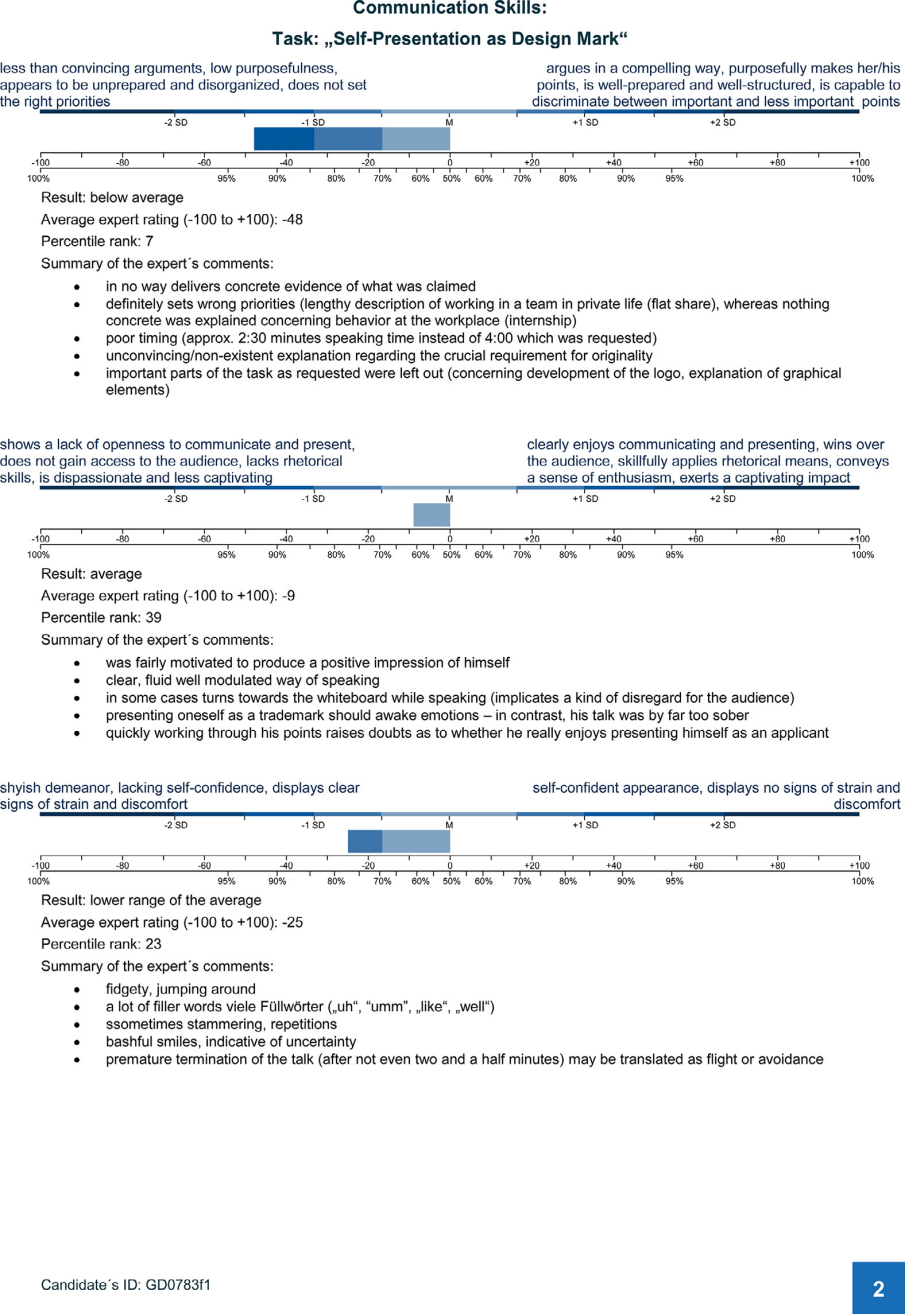
Document II: Expert rating report, page 2.

The expert rating report summarizes the candidate's poor performance with figures, numbers, and critical comments (on two pages; see Figs. 3 and 4).

### Document III: “Things you should in any case communicate to your conversation partner”

Document III was meant as a reminder. It contained information to communicate and questions to ask during the conversation (see Table 10).

**Table 10:** Document III: Things you should in any case communicate to your conversation partner.

Page	Text
1	**III. List: “Things you should in any case communicate to your conversation partner”**Please remind your conversation partner about both target dimensions that are subject of the assessment. Provide feedback regarding **all six results** including mention of • **typical behavior** on the left and right of the bar diagram, and • **classification of the results** in reference to the whole group of candidates (e.g., “above average”) Give account for at least two of the **expert's comments** regarding the respective dimension.Ask the candidate the following question: **“Could you explain to me the extent to which these results meet your expectations and your experience with your strengths and weaknesses?”**Ask the candidate the following questions: **“What are the conclusions you draw from the results? Do you feel encouraged to strive for the desired position? Alternatively, did the results encourage you to swing towards another goal?”**

### Document IV: “Directions and guidelines on conversation techniques”

Document IV summarized directions and guidelines on conversation techniques. As these directions referred to specific emotion regulation strategies, one out of the four versions of document IV was handed out according to the actual experimental condition (see Table 11).

**Table 11:** *Four versions of document IV:* “Directions and guidelines on conversation techniques”.

Experimental Condition	Text of Document IV
Expressive Suppression	**Retain a neutral facial expression so that nobody can observe any emotion!** Dissemble any feelings you may have. Do not respond to possible emotional expressions on the part of your conversation partner. Do not express any emotions. Keep your facial muscles motionless to retain a neutral expression. Please use phrases like “we,” “our team,” and “our raters”; do not distance yourself.
Cognitive Reappraisal	**Adopt the neutral and distanced attitude of a professional assessor and provide as much factual information as possible!** Try to think about the conversation in such a way that no emotions build up. Do not regard the feedback situation as being a personal matter; think about the conversation objectively and analytically. Always focus on the facts, pay attention to the details of the expert rating sheet, and express as many details as possible. State additional facts like the average expert rating (from −100 to +100) or the percentile rank in order to provide informative and objective feedback to your conversation partner. Always remember: “The most important thing about career choices is to know as much as possible about oneself – with a focus on both positive and negative aspects.” Please use phrases like “we,” “our team,” and “our raters”; do not distance yourself.
Affect Utilization	**Pay attention to all emotions that potentially arise during the conversation and make yourself an “expert” in every mood and emotion!** Get involved with your own feelings as well as the feelings of your conversation partner; put yourself in your conversation partner's position. Someone watching you should recognize that you have noticed any emotional nuance the candidate has expressed. Lend a sympathetic ear (nodding, eye contact, “mhm, yes”) Restate your conversation partner's remarks in your own words. Bring up feelings directly; tell your conversation partner what emotion you have discovered behind her or his statement (use an interrogative form). Please use phrases like “we,” “our team,” and “our raters”; do not distance yourself.
Control	**Conduct the conversation in a way that is most comfortable for you personally.** No strategy is better or worse than any other. Please use phrases like “we,” “our team,” and “our raters”; do not distance yourself.

## Manipulation check and results

Burk and Wiese (2021) have applied the protocol to 150 participants. As the central result, providing negative feedback was followed by significant temporary testosterone decreases as well as cortisol increases. In addition, testosterone (but not cortisol) responses were attenuated when feedback providers had been instructed to either follow a cognitive reappraisal or affect utilization strategy.

### Subjectively reported applicability of emotion regulation strategies

During the follow-up phase, participants were asked to provide details about their impression of the applicability of the instructions they had received. Descriptive statistics regarding the answers to three questions are given in Table 12. Altogether, participants reported a high comprehensibility of the instructions about how to behave during the feedback conversation. There were no substantial differences in the ratings between the four conditions. Asked about their impression how well they had been able to implement the instructions, participants in general reported moderate values. Implementing instructions to apply affect utilization have been perceived less feasible compared with instructions to apply cognitive reappraisal strategies. In addition, participants reported that the control condition's instructions were less useful to feel better than those of the cognitive reappraisal condition were.

**Table 12:** Means and standard deviations (in brackets) of participants’ rating regarding the applicability of the instructions.

Questions regarding Applicability	Experimental Conditions			
	Expressive Suppression	Cognitive Reappraisal	Affect Utilization	Control Condition
Comprehensibility of the instructions how to behave during the conversation (from 1 = “very incomprehensible” to 100 = “very well comprehensible”)	78.83 (23.32)	73.76 (21.52)	77.12 (23.03)	80.82 (19.10)
Feasibility to follow the instructions (from 1 = “I have been able to implement the instructions very poorly to 100″ = “I have been able to implement the instructions very well”)	50.44 (25.07)	54.09^a)^ (20.14)	37.19^b)^ (22.42)	42.47 (26.84)
Usefulness of the instructions to feel better (from 1 = “very useless to feel better” to 100 = “very useful to feel better”)	56.33 (26.78)	65.18^a)^ (20.02)	55.94 (26.38)	47.24^b)^ (30.41)

*Note.*^a)^ significantly more pronounced than ^b)^ in bonferroni-adjusted post-hoc tests.

### Self-Descriptions and observer ratings of positive and negative affect

Self-descriptions of affect across five measurement occasions were collected through the 20-item Positive and Negative Affect Schedule (PANAS; Watson, Clark, and Tellegen, 1988). To obtain simple, baseline adjusted mood response measures, PANAS self-description scores were specified as intercept and latent basis factors across five measurement occasions. Altogether, twelve out of 20 PANAS items revealed significant change over time. A clear curvilinear trajectory was obtained for seven mood items. On Average, participants reported a temporary increase of being distressed, upset, guilty, scared, less enthusiastic, ashamed, and jittery with a peak response immediately after the feedback conversation ended. In addition, there was an approximately linear decrease of being excited, strong, inspired, determined, and attentive across the entire session. Among these mood responses, solely the degree of feeling guilty right after the conversation was associated with a hormonal response: The stronger the decrease in T, the stronger was the increase regarding guilt.

Valid video recordings of the feedback conversations could be obtained in 115 cases. Based on these, ratings regarding 20 PANAS dimensions (Watson et al., 1988) were collected from three observers (undergraduate students with major in psychology) each. As a manipulation check, based on the video recordings of the conversation segment, two blinded observers rated the probabilities in percent of the respective participant belonging to the expressive suppression, cognitive reappraisal, and affect utilization groups, respectively. The two-way mixed single-score intraclass correlation for absolute agreement was good (*ICC* = 0.81) for the ratings of affect utilization. However, agreement between observers was less pronounced when it came to recognizing the cognitive appraisal (*ICC* = 0.56) or expressive suppression (*ICC* = 0.59) conditions. Regressing the mean observer ratings on the actual assignment to a certain condition revealed significant distinguishability in all conditions. Again, the affect utilization condition was recognized as such most clearly (mean probability score: 79.50; *SD* = 19.38). The cognitive appraisal (*M* = 56.41; *SD* = 20.86) and expressive suppression (*M* = 47.73; *SD* = 26.84) conditions were detected significantly better than random, too. However, these conditions turned out to be less distinguishable compared to the affect utilization condition. Taken together, observational data suggests that participants actually adjusted their observable behavior according to the experimental condition to which they had been assigned.

Connecting hormone responses with mean PANAS observer ratings, the amount of the testosterone change was negatively related with appearing strong and enthusiastic. The stronger the cortisol response, the more participants were rated as nervous, jittery, and afraid and the lesser they were rated as interested, strong, and inspired.

## Conclusion and implications for future research

Our central aim was to create a work-related laboratory protocol for investigating work-related stress that reliably triggers testosterone and cortisol responses. As a first result, general temporary testosterone decreases as well as cortisol increases in response to anticipating and conducting a demanding feedback conversation were detected (Burk and Wiese, 2021). In addition, the amount of testosterone decreases was differentially affected by the instruction how to regulate emotions during the conversation.

As the present form of the protocol only addresses negative feedback, future research might decide to incorporate positive and /or neutral feedback conditions by introducing AC applicants displaying high/average performances. This could further our knowledge about whether hormones specifically respond to the demands of providing negative feedback or to conducting a feedback conversation in general.

Regarding the precise meaning of hormonal responses, one has to keep the delayed availability of hormones in the salivary fluid into account. Still, we cannot precisely distinguish between reactions to anticipating and actually conducting a feedback conversation. Future research may introduce more frequent and/or instant hormone measures with the use of hormone sampling in blood. Alternatively, one may decide to separate the anticipation from the conversation phase by adapting the protocol (e.g. introducing an anticipation-only condition).

## Declaration of Competing Interest

The authors declare that they have no known competing financial interests or personal relationships that could have appeared to influence the work reported in this paper.

## References

[1] Burk C.L., Wiese B.S. (2021). How to alleviate the agony of providing negative feedback: emotion regulation strategies affect hormonal stress responses to a managerial task. Horm. Behav..

[2] Gross J.J. (1998). Antecedent- and response-focused emotion regulation: divergent consequences for experience, expression, and physiology. J. Pers. Soc. Psychol..

[3] Watson D., Clark L.A., Tellegen A. (1988). Development and validation of brief measures of positive and negative affect: the PANAS scales. J. Pers. Soc. Psychol..

